# *Xenorhabdus aichiensis* sp. nov., *Xenorhabdus anantnagensis* sp. nov., and *Xenorhabdus yunnanensis* sp. nov., Isolated from *Steinernema* Entomopathogenic Nematodes

**DOI:** 10.1007/s00284-023-03373-2

**Published:** 2023-07-26

**Authors:** Ricardo A. R. Machado, Aashaq Hussain Bhat, Carlos Castaneda-Alvarez, Tarique Hassan Askary, Vladimir Půža, Sylvie Pagès, Joaquín Abolafia

**Affiliations:** 1 Experimental Biology Research Group, Institute of Biology, University of Neuchâtel, Rue Emile-Argand 11, 2000 Neuchâtel, Switzerland; 2 Department of Biosciences, University Center for Research and Development, Chandigarh University, Mohali, Punjab, India; 3 Departamento de Sanidad Vegetal, Facultad de Ciencias Agronómicas, Universidad de Chile, Santiago, Chile; 4 Division of Entomology, Faculty of Agriculture, Sher-e-Kashmir University of Agricultural Sciences and Technology, Wadura Campus, Jammu, Jammu and Kashmir India; 5 Biology Centre CAS, Institute of Entomology, České Budějovice, Czech Republic; 6 INRAe, Université de Montpellier, Montpellier, France; 7 Departamento de Biología Animal, Biología Vegetal y Ecología, Universidad de Jaén, Campus ‘Las Lagunillas’, Jaén, Spain

## Abstract

Three bacterial strains, XENO-2^T^, XENO-7^T^, and XENO-10^T^, isolated from *Steinernema* entomopathogenic nematodes, were found to represent novel *Xenorhabdus* species. In this study, we describe these new species by whole-genome and whole-proteome phylogenomic reconstructions, by calculating sequence identity scores using core genome sequences, and by phenotypic characterization. Phylogenomic reconstructions using ribosomal and house-keeping genes, and whole-genome and whole-proteome sequences show that XENO-2^T^ and XENO-10^T^ are closely related to *Xenorhabdus japonica* DSM 16522^T^ and that XENO-7^T^ is closely related to *Xenorhabdus bovienii* subsp. *africana* XENO-1^T^ and to *X. bovienii* subsp. *bovienii* T228^T^. The dDDH values between XENO-2^T^ and XENO-10^T^ and between XENO-2^T^ and *X. japonica* DSM 16522^T^ are 56.4 and 51.8%, respectively. The dDDH value between XENO-10^T^ and *X. japonica* DSM 16522^T^ is 53.4%. The dDDH values between XENO-7^T^ and *X. bovienii* subsp. *africana* XENO-1^T^ and between XENO-7^T^ and *X. bovienii* subsp. *bovienii* T228^T^ are 63.6 and 69.4%, respectively. These dDDH values are below the 70% divergence threshold for prokaryotic species delineation. The newly described species are highly pathogenic to *G. mellonella* larvae, grow at pH between 5 and 9 (optimum 5–7), at salt concentrations of 1–3% (optimum 1–2%), and temperatures between 20 and 37 °C (optimum 28–30 °C). Biochemical tests such as lysine decarboxylase, ornithine decarboxylase, urease, gelatinase, citrate utilization, indole and acetoin production, and cytochrome oxidase tests allow to differentiate the novel species from their more closely related species. Considering these genetic and phenotypic divergencies, we propose the following new species: *Xenorhabdus aichiensis* sp. nov. with XENO-7^T^ (= CCM 9233^T^ = CCOS 2024^T^) as the type strain, *Xenorhabdus anantnagensis* sp. nov., with XENO-2^T^ (= CCM 9237^T^ = CCOS 2023^T^) as the type strain, and *Xenorhabdus yunnanensis* sp. nov., with XENO-10^T^ (= CCM 9322^T^ = CCOS 2071^T^) as the type strain. Our study contributes to a better understanding of the biodiversity and phylogenetic relationships of entomopathogenic bacteria associated with insect parasitic nematodes.

**Supplementary Information:**

The online version contains supplementary material available at 10.1007/s00284-023-03373-2.

## Introduction

Species of the bacterial genus *Xenorhabdus* establish a symbiotic association with *Steinernema* entomopathogenic nematodes (EPNs) [1]. *Steinernema* EPNs are soil-living organisms which parasitize and reproduce inside insects and certain other small arthropods [1]. These nematodes carry *Xenorhabdus* bacteria inside a specialized vesicle, which is located at the anterior part of the intestine of infective juveniles, and release them immediately after colonizing a host. *Xenorhabdus* bacteria produce different digestive enzymes, toxins, immunosuppressors, and antibiotics, which serve to kill and pre-digest the infected host [2]. Then, nematodes and bacteria proliferate in the cadaver [1]. Upon resource depletion, nematodes and bacteria re-establish their symbiosis and abandon the cadaver in search of a new host. Due to their remarkable ability to search for and kill insects, including crop pests, and due to the enormous biochemical capabilities of *Xenorhabdus* bacteria, these organisms are of high agricultural, biotechnological, and medical relevance [3].

The genus *Xenorhabdus* was created by Thomas and Poinar to accommodate large, gram-negative, rod-shaped, facultatively anaerobic, entomopathogenic bacteria which establish an obligated symbiosis with entomogenous nematodes [4]. Initially, it contained species of bacteria associated with *Steinernema* and with *Heterorhabditis* nematodes. Later, the creation of the genus *Photorhabdus* was proposed, and the symbiotic bacteria associated with *Heterorhabditis* nematodes, were transferred to this new genus [5, 6]. At that time, the taxonomic decisions were made mainly based on wet-lab DNA-DNA hybridization techniques and biochemical tests [7–11]. A few years later, the use 16S rRNA gene sequences was introduced [12] and successfully used to describe several novel species of the genus *Xenorhabdus* [13–15]. Shortly after, the use of multiple house-keeping genes for taxonomic purposes was introduced for the first time [16], which significantly improved the robustness of the phylogenetic reconstructions, and again led to the description of several novel species [17–19]. Boosted by the advances in genome sequencing techniques, the use of core genome sequences and in silico DNA-DNA hybridization techniques are the gold standard nowadays [20–22]. After decades of efforts to understand the biodiversity of this agriculturally and biotechnologically relevant group of bacteria, several species have been described. Currently, this genus contains 28 taxa with validly published names: 27 species and 1 subspecies [4, 10, 11, 13–26]. The complete list of species is found under: https://lpsn.dsmz.de/genus/xenorhabdus [26].

In this study, we phenotypically and molecularly characterized several bacterial strains, isolated from different species of *Steinernema* entomopathogenic nematodes, which represent new species in the genus *Xenorhabdus* for which we propose the following names: *Xenorhabdus aichiensis* sp. nov., *Xenorhabdus anantnagensis* sp. nov., and *Xenorhabdus yunnanensis* sp. nov. Our study contributes to a better understanding of the biodiversity of a bacterial group of biotechnological and agricultural relevance and thereby further advances our efforts toward developing more biocontrol tools for sustainable and environmentally friendly agriculture.

## Materials and Methods

### Strain Isolation and Biogeography

The three bacterial strains characterized in this study, XENO-2^T^, XENO-7^T^, and XENO-10^T^, were isolated from *Steinernema* entomopathogenic nematodes. XENO-2^T^ was isolated from a novel *Steinernema* nematode species, which will be described somewhere else [27]. These nematodes were isolated from soil samples collected in Anantnag (Jammu and Kashmir, India) (GPS coordinates: 33.828914, 75.100091) using *Corcyra cephalonica* Stainton (Lepidoptera: Pyralidae) larvae as baits. XENO-7^T^ was isolated from *S. litorale* nematodes. These nematodes were isolated from soil samples collected from a coastal pine forest (Cape Irago-zaki, Atsumi Peninsula, Aichi Prefecture, Honshu, Japan) using the *Galleria* baiting technique [28]. XENO-10^T^ was isolated from *S. akhursti* nematodes. These nematodes were isolated from soil samples collected from a grassland in the Cigu village (Deqen, Diqing Tibetan Autonomous Prefecture, Yunnan province, China) using *G. mellonella* larvae as baits [29]. Nematode identification was carried out as described below. To isolate the bacterial strains, *G. mellonella* larvae were infested with the different nematode strains. Three to four days later, several insect cadavers were surface-sterilized and dissected with a sterile surgical blade. Insect internal organs were spread onto Lysogeny Broth (LB) agar plates (Sigma-Aldrich, Switzerland) and incubated at 28 °C for 48-96 h. *Xenorhabdus*-like colonies were subcultured until monocultures were obtained [30]. Different procedures, such as the characterization of colony and cell morphology, and 16S rRNA gene sequencing were carried out to determine the culture purity.

### Bacterial 16S rRNA Gene Phylogeny and Sequence Comparisons

To determine the taxonomic identities of XENO-2^T^, XENO-7^T^, and XENO-10^T^, genomic DNA was first extracted and purified from bacterial monocultures using the GenElute Bacterial Genomic DNA Kit (Sigma–Aldrich, Switzerland) following the manufacturer’s instructions. The resulting DNA was used to obtain 16S rRNA gene sequences by polymerase chain reaction (PCR) using the following universal primers: 27F (5′-AGAGTTTGATCMTGGCTCAG-3′) and 1525R (5′-AAGGAGGTGWTCCARCC-3′) and the following cycling conditions: 1 cycle at 94 °C for 10 min followed by 40 cycles at 94 °C for 30 s, 55 °C for 30 s, and 72 °C for 60 s, followed by a final extension at 72 °C for 5 min as described previously [31–33]. PCR products were separated by electrophoresis in a 1% TAE-agarose gel stained with GelRed nucleic acid gel stain (Biotium), gel-purified (QIAquick Gel Purification Kit, Qiagen), and sequenced by Sanger sequencing (Microsynth AG, Balgach, Switzerland). The resulting 16S rRNA raw sequences were manually curated using Bioedit 7.2.5 [34]. In addition, 16S rRNA gene sequences were obtained directly from whole-genome sequences using the bacterial ribosomal RNA predictor Barrnap 0.7 using the following parameters: reject length threshold = 0.5; length cutoff = 0.8; and *e*-value = 0.00001 [35]. The obtained sequences were identical to those obtained by Sanger sequencing. 16S rRNA gene-based phylogenetic relationships were reconstructed using the Maximum Likelihood method based on the Kimura 2-parameter model in MEGA7 [36–38]. To this end, sequences were aligned with MUSCLE (v3.8.31) [39]. The tree with the highest log likelihood was obtained. The percentage of trees in which the associated taxa clustered together is shown next to the branches. Initial tree(s) for the heuristic search were calculated automatically by applying Neighbor-Join and BioNJ algorithms to a matrix of pairwise distances estimated using the Maximum Composite Likelihood (MCL) approach, and then selecting the topology with superior log likelihood value. The tree is drawn to scale, with branch lengths measured in the number of substitutions per site. Graphical representation and edition of the phylogenetic trees were performed with Interactive Tree of Life (v3.5.1) [40, 41].

### Recombinase A Gene-Based Phylogenetic Reconstructions

Recombinase A (*recA*) gene sequences were obtained directly from the whole-genome sequences using BLAST. The obtained sequences were used to reconstruct phylogenetic relationships using the maximum likelihood method based on the Kimura 2-parameter model in MEGA7 as described above [36–38]. Graphical representation and edition of the phylogenetic trees were performed with Interactive Tree of Life (v3.5.1) [40, 41].

### Genome Sequencing and Genomic Features

The genomes of the following strains were obtained in this study: *X. aichiensis* sp. nov. XENO-7^ T^, *X. anantnagensis* sp. nov. XENO-2^T^, *X. griffiniae* ID10^T^, *X. khoisanae* SF87^T^, *X. magdalenensis* IMI 397775^T^, *X. romanii* PR06-A^T^, and *X. yunnanensis* sp. nov. XENO-10^T^. Genomes sequences were obtained as described previously [42, 43]. Briefly, genomic DNA was extracted and purified using the GenElute Bacterial Genomic DNA Kit (Sigma–Aldrich, Switzerland) following the manufacturer’s instructions. The resulting DNA was used for library preparation using the TruSeq DNA PCR–Free LT Library Prep (FC–121–3003) kit. Indexed libraries were then pooled at equimolar concentrations and sequenced (2 × 150 bp) on an Illumina HiSeq 3000 instrument. Genomes were assembled using the Bactopia pipeline [44]. To this end, the raw Illumina reads were quality trimmed using Trimmomatic 0.39 (options: slidingwindow:4: 8, minlen:127) [45]. The resulting reads were assembled with SPAdes 3.14.1 (-careful, -mismatch-correction, k–mer sizes of 31, 51, 71, 91, and 111 bp) [46]. Scaffolds with a mean read–depth smaller than 20% of the median read–depth of the longer scaffolds (≥ 5000 bp) as well as scaffolds that were shorter than 200 bp were removed. Minor assembly errors were corrected using Pilon 1.22 with default parameters [47]. Completeness and contamination of the assembled genomes were assessed using checkM v1.1.6 with default parameters [48].

### Core Genome- and Core Proteome-Based Phylogenetic Reconstructions and Sequence Comparisons

To reconstruct whole-genome-based phylogenetic relationships, genomes were first aligned using Roary 3.13.0. Genes to be considered core should be presented in 85% of the genomes with an 85% protein identity, and a coverage higher than 90%. Obtained alignments were used to build phylogenomic trees using FastTree 2.1.10 based on the Generalized Time Reversible Model (GTR). Branch support was assessed using the Shimodaira-Hasegawa-like procedure based on 100 replicates. To reconstruct whole-proteome-based phylogenetic relationships, first, all ORFs from all genomes were extracted using Prodigal [49]. Then, homologous genes (80% or higher similarity and a coverage higher than 90%) were clustered using MMSEQS2 (e-value: 0.001, sensitivity: 7.5, and cover: 0.5) and MCL (Inflation = 2) [50–52]. Orthologous genes were then translated and aligned using MAFFT [53]. Orthologous genes were considered core genes if they were present in more than 80% of the genomes analyzed. Lastly, a maximum likelihood-based phylogenetic tree was reconstructed based on the inferred core-proteome alignment using RAxML [54, 55]. Branch support was assessed using the rapid bootstrap method based on 100 replicates [56]. Graphical representation and edition of the phylogenetic trees were performed with Interactive Tree of Life (v3.5.1) [40, 41]. Whole-genome sequence similarities were calculated by the GBPD (Genome Blast Distance Phylogeny) method using the Genome-to-Genome Distance Calculator 2.1 and formula 2 of the Deutsche Sammlung von Mikroorganismen und Zellkulturen (DSMZ) web service (http://ggdc.dsmz.de) using default parameters [57–60].

### Physiological, Biochemical, and Morphological Characterization

To physiologically, biochemically and morphologically characterize the newly isolated bacterial strains, bacterial cultures from single primary form colonies were used. Bacterial primary forms were determined by examining their ability to absorb dye from NBTA culturing plates (LB agar plates supplemented with 25 mg L^−1^ bromothymol blue and 4 mg L^−1^ triphenyl-2,3,5-tetrazolium chloride) or by their ability to produce yellow, orange, and reddish pigments. The selected primary form colonies were further subcultured and maintained on Lysogeny Broth (LB) agar plates at 28–30 °C. Cell morphology was observed under a Leica DM4B optical microscope at ×1000 magnification, with cells grown for 16 h at 28 °C on LB. Light microscopy photographs were captured using a Leica DM4 B optical microscope equipped with a Leica DFC 7000T Camera (Leica, Wetzlar, Germany). The optimum temperature for bacterial growth was evaluated on regular LB agar medium (pH 7, 1% NaCl) at 20 °C, 24 °C, 28 °C, 30 °C, 37 °C, and 42 °C. Growth on medium containing different salt concentrations was evaluated in 3 mL of LB medium (pH 7) in 15 mL Falcon tubes. Three NaCl concentrations were used: 1% (Regular LB medium), 2%, and 3%. Growth at different pH was evaluated in 3 mL of LB medium (1% NaCl) in 15 mL Falcon tubes. Five different pH were used: 3, 5, 7 (Regular LB medium), 8, and 9. Each tube was inoculated with 0.1 mL of an overnight bacterial culture, then incubated for 24 h at 28 °C and 180 rpm. Three tubes per treatment were considered. Antibiotic resistance was evaluated on regular LB medium (pH 7, 1% NaCl) containing either tetracycline, vancomycin, or gentamicin at concentrations of 30 mg/L. Cytochrome oxidase production was tested on disks containing *N,N*-dimethyl-p-phenylenediamine oxalate and α-naphthol (Sigma-Aldrich, Switzerland). Catalase activity was determined by adding a drop of 10% (v/v) H_2_O_2_ into 50 µL of a 16 h-old liquid LB-bacterial culture. The ability to absorb dye was tested by growing the cells on NBTA agar. Biochemical characterization was carried out using the API20E system (bioMérieux, Inc. Durham, NC) according to the manufacturer’s instructions. To this end, bacteria were grown for 16 h at 28 °C in LB agar Petri plates. Then, one single colony was re-suspended in 5 mL of 0.85% NaCl. The resulting bacterial solution was used to inoculate the different microtubes containing the biochemical tests. Samples were incubated at 28 °C. Results were evaluated after 24 h. Gram staining was carried out using the Gram-Color modified (phenol-free) staining kit following the manufacturer’s instructions (Sigma–Aldrich, Switzerland). Cell morphology, optimum temperature for bacterial growth, growth on medium containing different salt concentrations and pH, and Gram reaction were evaluated only in the novel species described in this study. Biochemical tests such as cytochrome oxidase production, catalase activity, and the API20E tests were evaluated, in parallel, two independent times, in all the eight strains as listed in Table 1.

**Table 1:** Phenotypic characters of the type strains of different *Xenorhabdus* species

	1	2	3	4	5	6	7	8
β-Galactosidase	−	−	−	−	−	−	−	−
Arginine dihydrolase	+	+	+	+	+	+	+	+
Lysine decarboxylase	−	+	−	+	−	−	+	−
Ornithine decarboxylase	−	+	−	−	−	−	+	−
Citrate utilization	+	+	−	+	+	+	+	−
H_2_S production	−	−	−	−	−	−	−	−
Urease	−	+	−	+	−	−	+	−
Tryptophan deaminase	−	−	−	−	−	−	−	−
Indole production	−	+	−	−	−	−	−	+
Acetoin production	+	−	+	+	+	+	+	+
Gelatinase	+	+	−	−	−	+	+	+
Glucose oxidation	+	+	+	+	+	+	+	+
Mannitol oxidation	−	−	−	−	−	−	−	−
Inositol oxidation	−	−	−	−	−	−	−	−
Sorbitol oxidation	−	−	−	−	−	−	−	−
Rhamnose oxidation	−	−	−	−	−	−	−	−
Sucrose oxidation	−	−	−	−	−	−	−	−
Melibiose oxidation	−	−	−	−	−	−	−	−
Amygdalin oxidation	−	−	−	−	−	−	−	−
Arabinose oxidation	−	−	−	−	−	−	−	−
(Cytochrome) oxidase	+	+	+	+	+	−	+	−
Catalase	−	−	−	−	−	−	−	−
NO_2_ production	−	−	−	−	−	−	−	−
NO_2_ reduction to N_2_ gas	−	−	−	−	+	−	−	+

1: *X. aichiensis* sp. nov. XENO-7^T^; 2: *X. anantnagensis* sp. nov. XENO-2^T^; 3: *X. bovienii* subsp. *africana* XENO-1^T^; 4: *X. bovienii* subsp. *bovienii* T228^T^; 5: *X. japonica* DSM 16522^T^; 6: *X. poinarii* G6^T^; 7: *X. vietnamensis* VN01^T^; 8: *X. yunnanensis* sp. nov. XENO-10^T^

+  positive reaction, − negative reaction

### Genomic Comparative Analyses

Genomic comparative analyses to annotate and determine the presence/absence of genes that are involved in antibiotic resistance or in the production of specialized metabolites were carried out by aligning draft genome assemblies or predicted proteomes against The Comprehensive Antibiotic Resistance Database (“CARD”) [61–66] and against the antibiotics and secondary metabolite analysis shell (antiSMASH) database [67, 68]. Genes that passed the threshold values (antibiotic resistance: ≥ 70% nucleotide identity and ≥ 50% coverage; and antiSMASH: ≥ 50% nucleotide identity) were considered as present in the genome. Below these thresholds, genes were considered absent or non-functional.

### Ecological Characterization

*Xenorhabdus* bacteria are characterized by their ability to produce different insecticidal molecules [3]. To evaluate the entomopathogenic potential of the novel species, bacteria were cultured overnight in LB liquid medium. Then, the bacterial cultures were collected and their optical densities at 600 nm (OD_600_) were measured. All cultures were then diluted to reach an OD_600_ = 1. The resulting cultures were further diluted to obtain four bacterial solutions with OD_600_= 0.1, 0.25, 0.5, or 1. Then, 10 µL of the resulting bacterial solutions were injected into third-instar *G. mellonella* larvae. Ten larvae per bacterial strain and dilution were injected (*n* = 10). Mortality was evaluated at 48 and 72 h after injections. Results were statistically assessed by repeated-measures ANOVA with bacterial strain and time as factors. Normality and equality of variance were verified using Shapiro–Wilk and Levene's tests, respectively. Holm–Sidak post hoc tests were used for multiple comparisons. All statistical analyses were conducted using Sigma Plot 14.5 (Systat Software Inc., San Jose, CA, USA).

### Nematode Molecular Characterization and Phylogenetic Analyses

The nematodes that host the novel bacterial species described in this study were molecularly identified based on rRNA gene sequences [69]. To extract genomic DNA from single virgin females, several individuals were collected and washed with Ringer’s solution, then with PBS buffer, and then transferred individually into sterile PCR tubes (0.2 mL), each containing 20 μL extraction buffer (17.6 μL nuclease-free dH_2_O, 2 μL 5X PCR buffer, 0.2 μL 1% Tween, and 0.2 μL proteinase K). Samples were then frozen at − 20 °C for 60 min and then incubated in a water bath at 65 °C for 1 h, and at 95 °C for 10 min. The lysates were incubated on ice for 20 min and centrifuged at 6500 g for 2 min. No further DNA purification was carried out and the supernatants were directly used for PCRs. To amplify the internal transcribed spacer regions (ITS1-5.8S-ITS2), the following primers were used: 18S: (5′-TTGATTACGTCCCTGCCCTTT-3′) (forward), and 28S: (5′-TTTCACTCGCCGTTACTAAGG-3′) (reverse) [70]. The 25 µL PCRs consisted of 12.5 µL of Dream Taq Green PCR Master Mix (Thermo Scientific, USA), 0.5 µL of each forward and reverse primer at 10 µM, 2 µL of DNA extract, and 9.5 µL of nuclease-free distilled water. The PCR was performed using a thermocycler with the following settings: 1 cycle of 5 min at 94 °C followed by 37 cycles of 30 s at 94 °C, 30 s at 50 °C, 1 min 30 s at 72 °C, and by a single final elongation step at 72 °C for 10 min. PCR products were separated by electrophoresis (40 min, 130 V) in a 1% TBA (Tris–boric acid–EDTA)-buffered agarose gel stained with SYBR Safe DNA Gel Stain (Invitrogen, Carlsbad, California, USA). PCR products were purified using QIAquick PCR Purification Kit (Qiagen, Valencia, CA) and sequenced using reverse and forward primers by Sanger sequencing (Bioserve Ltd., Hyderabad, India). The obtained sequences were manually curated and trimmed using BioEdit, and deposited in the NCBI. To obtain sequences of closely related nematodes, we searched the database of NCBI by the Basic Local Alignment Search Tool (BLAST) [71]. The resulting sequences were used to reconstruct phylogenetic relationships by the maximum likelihood method based on Hasegawa-Kishino-Yano model (HKY+G) nucleotide substitution model as described above. To select the best substitution model, best–fit nucleotide substitution model analyses were conducted in MEGA 7 [38, 72].

## Results and Discussion

### Bacterial 16S rRNA Gene-Based Phylogeny and Sequence Comparisons

Phylogenetic reconstructions based on 16S rRNA gene sequences show that *X. anantnagensis* sp. nov. XENO-2^T^ and *X. yunnanensis* sp. nov. XENO-10^T^ form a unique clade together with *X. japonica* DSM 16522^T^ and *X. vietnamensis* VN01^T^ (Fig. S1, Table S1). In turn, *X. aichiensis* sp. nov. XENO-7^T^ forms a unique clade together with *X. bovienii* subsp. *africana* XENO-1^T^ and *X. bovienii* subsp. *bovienii* T228^T^ (Fig. S1). 16S rRNA gene sequence similarity scores between *X. anantnagensis* sp. nov. XENO-2^T^ and *X. vietnamensis* VN01^T^, and between *X. anantnagensis* sp. nov. XENO-2^T^ and *X. japonica* DSM 16522^T^ are 98.9% in both cases (Fig. S2). 16S rRNA gene sequence similarity scores between *X. yunnanensis* sp. nov. XENO-10^T^ and *X. vietnamensis* VN01^T^, and between *X. yunnanensis* sp. nov. XENO-10^T^ and *X. japonica* DSM 16522^T^ are 98.3% in both cases. The 16S rRNA gene sequence similarity score between *X. anantnagensis* sp. nov. XENO-2^T^ and *X. yunnanensis* sp. nov. XENO-10^T^ is 98.7%. Lastly, 16S rRNA gene sequence similarity scores between *X. aichiensis* sp. nov. XENO-7^T^ and *X. bovienii* subsp. *africana* XENO-1^T^, and between *X. aichiensis* sp. nov. XENO-7^T^ and *X. bovienii* subsp. *bovienii* T228^T^ are 99.2 and 99.4%, respectively. Lower sequence similarities were observed when the sequences of *X. anantnagensis* sp. nov. XENO-2^T^, *X. aichiensis* sp. nov. XENO-7^T^, and *X. yunnanensis* sp. nov. XENO-10^T^ were compared to the sequences of the type strains of all the species of the genus *Xenorhabdus* with validly published names (Fig. S2)*.* 16S rRNA gene sequences are deposited in the National Center for Biotechnology Information (NCBI) databank under the accession numbers OQ439938, OQ439939, and OQ439940, respectively.

### Bacterial Recombinase A Gene-Based Phylogeny

Phylogenetic reconstructions based on the recombinase A (*recA*) gene sequences show that *X. anantnagensis* sp. nov. XENO-2^T^ and *X. yunnanensis* sp. nov. XENO-10^T^ form a unique clade together with *X. japonica* DSM 16522^T^ and *X. vietnamensis* VN01^T^ (Fig. S3). In turn, *X. aichiensis* sp. nov. XENO-7^T^ forms a unique clade together with *X. bovienii* subsp. *africana* XENO-1^T^ and *X. bovienii* subsp. *bovienii* T228^T^ (Fig. S3).

### Genome Sequencing and Genomic Features

The main characteristics of the genomes obtained in this study are summarized in Tables S2, S3, and S4. The main characteristics of the genomes of the novel species are as follows. The genome of *X. aichiensis* sp. nov. XENO-7^T^ contains 4,699,893 bp, a G+C content of 44.63%, and 4196 proteins (Tables S2, S3). The genome of *X. anantnagensis* sp. nov. XENO-2^T^ contains 4,318,764 bp, a G+C content of 42.88%, and 3905 proteins (Tables S2, S3). The genome of *X. yunnanensis* sp. nov. XENO-10^T^ contains 4,667,994 bp, a G+C content of 43.14%, and 4534 proteins (Tables S2, S3). These genomes are predicted to be more than 99% complete and contain less than 2% of contamination (Table S4). Whole-genome sequences of XENO-2^T^, XENO-7^T^, and XENO-10^T^ are deposited in the National Center for Biotechnology Information (NCBI) databank under the accession numbers JAQRFN01, JAQRFO01, and JAQRFI01, respectively.

### Core Genome- and Core Proteome-Based Phylogenetic Reconstructions and Sequence Comparisons

**Fig. 1:**
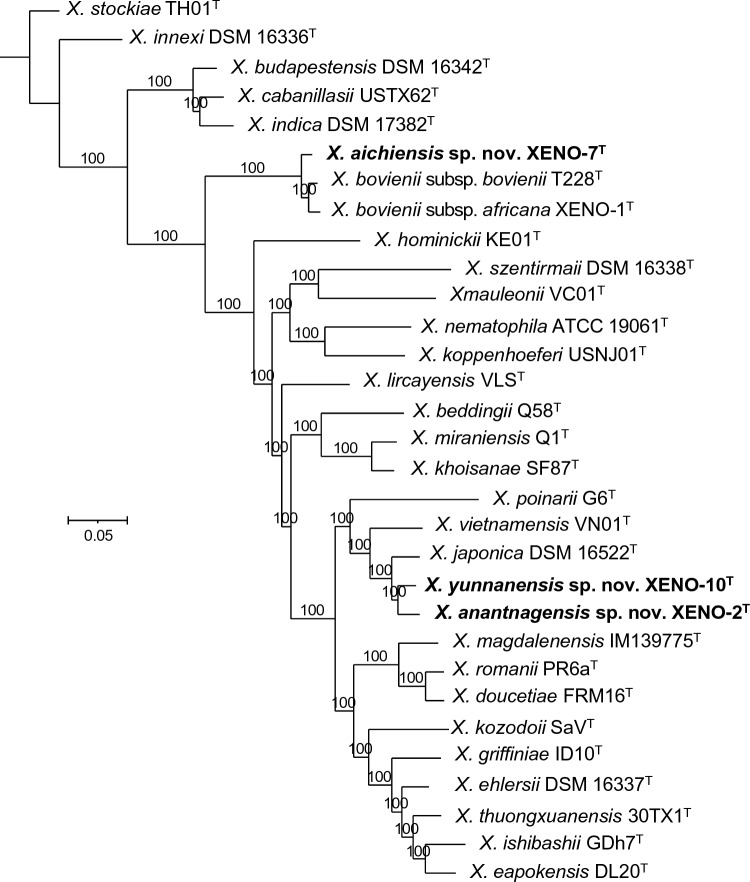
Phylogenetic reconstruction based on core genome sequences of *Xenorhabdus* bacterial strains. 1,485,300 nucleotide positions (1454 core genes) were used in the analysis. Numbers at the nodes represent SH-like branch supports. Bar represents 0.05 nucleotide substitutions per sequence position. The National Center for Biotechnology Information (NCBI) accession numbers of the sequences used for these analyses are shown in Table S1

**Fig. 2:**
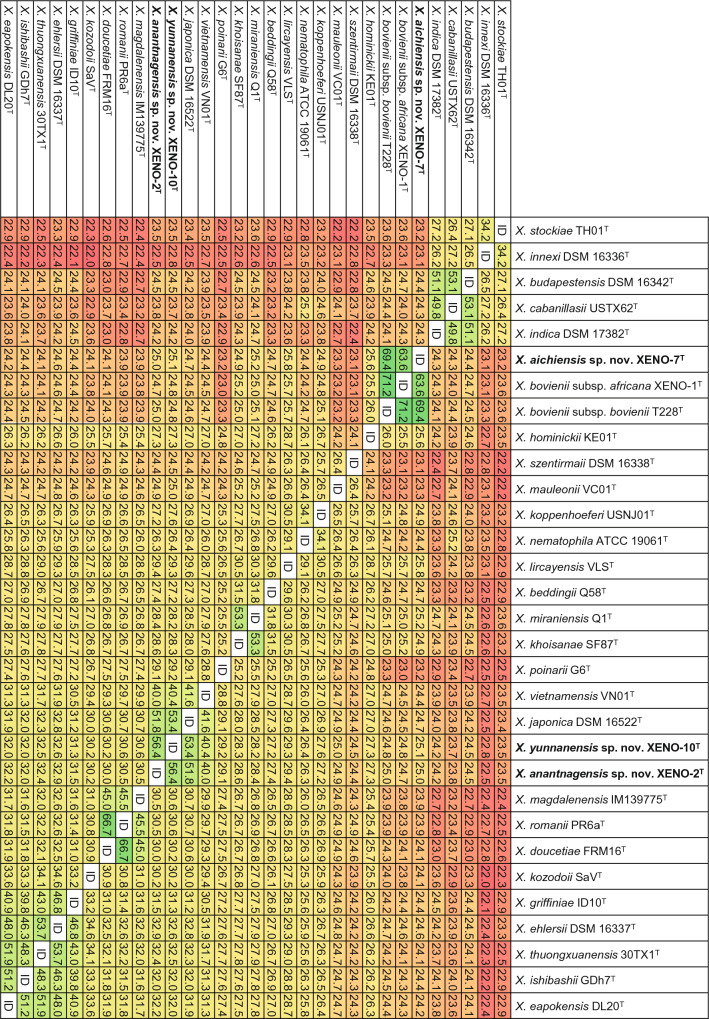
Pairwise comparison of digital DNA-DNA Hybridization (dDDH) scores (%) of *Xenorhabdus* strains. NCBI accession numbers of gene sequences used are shown in Table S1

Core-genome and core-proteome phylogenies show that *X. aichiensis* sp. nov. XENO-7^T^ forms a unique clade together with *X. bovienii* subsp. *africana* XENO-1^T^ and *X. bovienii* subsp. *bovienii* T228^T^ (Figs. 1, S4). In addition, *X. anantnagensis* sp. nov. XENO-2^T^ and *X. yunnanensis* sp. nov. XENO-10^T^ form a unique clade together with *X. vietnamensis* VN01^T^, *X. japonica* DSM 16522^T^, and *X. poinarii* G6^T^ (Figs. 1, S4). The dDDH values between XENO-7^T^ and *X. bovienii* subsp. *africana* XENO-1^T^, and between XENO-7^T^ and *X. bovienii* subsp. *bovienii* T228^T^ are 63.6 and 69.4%, respectively (Fig. 2). The dDDH values between XENO-2^T^ and XENO-10^T^, and between XENO-2^T^ and *X. japonica* DSM 16522^T^ are 56.4 and 51.8%, respectively. The dDDH value between XENO-10^T^ and *X. japonica* DSM 16522^T^ is 53.4%. Lower sequence similarities were observed when the sequences of *X. anantnagensis* sp. nov. XENO-2^T^, *X. aichiensis* sp. nov. XENO-7^T^, and *X. yunnanensis* sp. nov. XENO-10^T^ were compared to the sequences of the type strains of all the species of the genus *Xenorhabdus* with validly published names. Given that the observed dDDH values are below the 70% divergence threshold for prokaryotic species delineation, XENO-2^T^, XENO-7^T^, and XENO-10^T^ represent novel species within the genus *Xenorhabdus* [73].

### Physiological and Biochemical Characterization

Biochemical tests show that the three novel species exhibit biochemical capacities that are similar to the biochemical capacities of several members of the *Xenorhabdus* genus (Table 1). However, the three novel species also exhibit unique biochemical capacities that differ from the biochemical capacities of their most closely related species (Table 1)*.* In particular, lysine decarboxylase, citrate utilization, and urease and gelatinase activity allow to differentiate *X. aichiensis* sp. nov. XENO-7^T^, *X. bovienii* subsp. *africana* XENO-1^T^, and *X. bovienii* subsp. *bovienii* T228^T^. In addition, lysine decarboxylase, ornithine decarboxylase, citrate utilization, urease activity, indole and acetoin production, gelatinase activity, cytochrome oxidase, and NO_2_ reduction to N_2_ gas allow to differentiate *X. anantnagensis* sp. nov. XENO-2^T^, *X. yunnanensis* sp. nov. XENO-10^T^, *X. vietnamensis* VN01^T^, *X. japonica* DSM 16522^T^, and *X. poinarii* G6^T^ (Table 1).

### Antibiotic Resistance

The genomes of *X. aichiensis* sp. nov. XENO-7^T^, *X. anantnagensis* sp. nov. XENO-2^T^, and *X. yunnanensis* sp. nov. XENO-10^T^ and the genomes of their more closely related species contain several antibiotic resistance genes (Table 2). In vitro experiments confirm the predicted antibiotic resistance patterns of these strains (Table S5). In particular, *X. aichiensis* sp. nov. XENO-7^T^, *X. anantnagensis* sp. nov. XENO-2^T^, *X. yunnanensis* sp. nov. XENO-10^T^, *X. bovienii* subsp. *africana* XENO-1^T^, and *X. bovienii* subsp. *bovienii* T228^T^ are susceptible to tetracycline, and *X. japonica* DSM 16522^T^, *X. poinarii* G6^T^, and *X. vietnamensis* VN01^T^ are resistant to this antibiotic at the dose tested (Table S5). All these strains resist gentamicin, and only *X. bovienii* subsp. *africana* XENO-1^T^ was found vancomycin susceptible (Table S5).

**Table 2:** Antibiotic resistance conferring genes present in the genomes of *X. aichiensis* sp. nov. XENO-7^T^, *X. anantnagensis* sp. nov. XENO-2^T^, *X. yunnanensis* sp. nov. XENO-10^T^*,* and in the genomes of the type strains of their closest relative species

Gene	Resistance mechanism	AMR gene family	Drug class	*X. aichiensis* sp. nov. XENO-7^**T**^	*X. anantnagensis* sp. nov. XENO-2^**T**^	*X. bovienii* subsp. *africana* XENO-1^T^	*X. bovienii* subsp. *bovienii* T228^T^	*X. japonica* DSM 16522^T^	*X. poinarii* G6^T^	*X. vietnamensis* VN01^T^	*X. yunnanensis* sp. nov. XENO-10^**T**^
CRP	Antibiotic efflux	Resistance-nodulation-cell division (RND) antibiotic efflux pump	Macrolide, fluoroquinolone, and penam	+	+	+	+	+	+	−	+
rsmA			Fluoroquinolone, diaminopyrimidine, and phenicol	+	+	+	+	+	+	+	+
KpnH		Major facilitator superfamily (MFS) antibiotic efflux pump	Macrolide, fluoroquinolone, aminoglycoside, carbapenem, cephalosporin, penam, and penem	+	+	+	+	+	+	+	+
KpnF			Macrolide, aminoglycosides, cephalosporin, tetracycline, peptides, rifamycin, disinfecting agents and antiseptics	−	−	−	−	+	+	+	−
qacJ		Small multidrug resistance (SMR) antibiotic efflux pump	Disinfecting agents and antiseptics	+	−	+	+	−	−	+	−
vanT	Antibiotic target alteration	Glycopeptide resistance gene cluster	Glycopeptides	+	+	−	+	+	+	+	+
EF-Tu		Elfamycin-resistant EF-Tu	Elfamycin	+	−	−	−	−	+	+	−

(+): present; (−): absent or non-functional

### Genomic Comparative Analyses

At the functional level, according to the antibiotics and secondary metabolite analysis shell (antiSMASH) database, the genomes of *X. aichiensis* sp. nov. XENO-7^T^, *X. anantnagensis* sp. nov. XENO-2^T^, and *X. yunnanensis* sp. nov. XENO-10^T^, and the genome of their more closely relates species contain biosynthetic gene clusters dedicated to the production of several phenazines, aryl polyenes, polyketides, and non-ribosomal peptides such as 1,6-phenazinedimethanol, acinetobactin, ambactin, bicornutin A1-A2, cycloserine, fabclavine, glidopeptin, indigoidine, kleboxymycin, luminmide, nematophin, puromycin, putrebactin, pyrrolizixenamide A, ralsolamycin, rhizomide A-C, syringomycin, syringopeptin 25A, taxlllaid A, thiomarinol, turnerbactin, xefoampeptides AG, xenematide, xeneprotides A-C, xenoamicin A-B, xenocoumacin I-II, and xenortide A-D (Table 3). The production of several of these compounds occurs in a species-specific manner as not all the species analyzed contain the gene cluster necessary for their biosynthesis (Table 3). In particular, only *X. aichiensis* sp. nov. XENO-7^T^ contains the genes to produce kleboxymycin, Syringopeptin 25A, and Xeneprotides A-C; only *X. bovienii* subsp. *africana* XENO-1^T^ contains the genes to produce closerine and syringomycin; only *X. bovienii* subsp. *bovienii* T228^T^ contains the genes to produce glidopeptin and puromicin; and only *X. yunnanensis* sp. nov. XENO-10^T^ contains the genes to produce ralsolamycin (Table 3).

**Table 3:** Predicted specialized metabolites produced by *X. aichiensis* sp. nov. XENO-7^T^, *X. anantnagensis* sp. nov. XENO-2^T^, *X. yunnanensis* sp. nov. XENO-10^T^*,* and by the type strains of their closest relative species

	*X. aichiensis* sp. nov. XENO-7^**T**^	*X. anantnagensis* sp. nov. XENO-2^**T**^	*X. bovienii* subsp. *africana* XENO-1^T^	*X. bovienii* subsp. *bovienii* T228^T^	*X. japonica* DSM 16522^T^	*X. poinarii* G6^T^	*X. vietnamensis* VN01^T^	*X. yunnanensis* sp. nov. XENO-10^**T**^
Specialized metabolite								
1,6-phenazinedimethanol	−	+	−	−	+	−	+	+
Acinetobactin	−	−	−	−	+	+	−	−
Ambactin	+	−	+	+	−	−	−	−
Aryl polyenes	−	+	−	+	+	−	−	+
Bicornutin A1–A2	−	+	−	−	−	−	+	+
Cycloserine	−	−	+	−	−	−	−	−
Fabclavine	+	−	+	+	−	−	−	−
Glidopeptin	−	−	−	+	−	−	−	−
Indigoidine	−	−	−	−	+	+	−	−
Kleboxymycin	+	−	−	−	−	−	−	−
Luminmide	+	−	−	−	+	−	−	−
Nematophin	+	−	+	−	−	−	+	−
Puromycin	−	−	−	+	−	−	−	−
Putrebactin	+	−	+	+	−	−	−	−
Pyrrolizixenamide A	−	+	−	−	+	+	+	−
Ralsolamycin	−	−	−	−	−	−	−	+
Rhizomide A–C	−	+	−	+	+	+	−	+
Syringomycin	−	−	+	−	−	−	−	−
Syringopeptin 25A	+	−	−	−	−	−	−	−
Taxlllaid A	−	−	+	+	−	−	−	−
Thiomarinol	+	−	+	+	+	+	+	−
Turnerbactin	−	+	−	−	−	−	+	+
Xefoampeptides A–G	+	+	+	+	−	−	+	−
Xenematide	+	−	−	+	−	−	+	−
Xeneprotides A–C	+	−	−	−	−	−	−	−
Xenoamicin A–B	+	+	+	+	+	−	+	+
Xenocoumacin I–II	+	−	+	+	−	−	+	+
Xenortide A–D	+	−	+	−	−	−	−	−

(+): Produced; (−): Not produced

### Ecological Characterization

When injected into the hemocoel of *G. mellonella* larvae, all the strains evaluated in this study rapidly killed the insects in a bacterial density-dependent manner (Fig. S5). At the lowest bacterial density tested (OD_600_ = 0.1),  all the bacterial strains killed between 50 and 80% of the larvae in less than 48 h after injections, being *X. aichiensis* sp. nov. XENO-7^T^ and *X. anantnagensis* sp. nov. XENO-2^T^ the less pathogenic strains. More insects were killed when the bacterial densities injected were increased, and in some cases, even 100% of the insects were killed within 48 h. 72 h after injections, almost all insects were killed, especially when the bacterial densities injected were high (Fig. S5). Overall, *X. anantnagensis* sp. nov. XENO-2^T^ was apparently the less pathogenic strain, but they still killed more than 70% of the insects at the lowest bacterial density tested (Fig. S5).

### Nematode and Bacteria Co-phylogenies

To infer about potential co-evolutionary processes between the newly described bacterial species and their nematode hosts, we reconstructed phylogenetic relationships of these two groups of organisms and of closely related species and compared the resulting phylogenetic trees (Fig. S6). We observed very interesting patterns. Specifically, the nematode hosts of the following four closely related strains: *X. anantnagensis* sp. nov. XENO-2^T^, *X. yunnanensis* sp. nov. XENO-10^T^, *X. japonica* DSM 16522^T^, and *X. vietnamensis* VN01^T^ are *S. akhursti*, *Steinernema* sp., *S. kushidai*, and *S. sangi,* respectively. These nematode species are also closely related (Fig. S6). Similarly, the nematodes that host the following three closely related bacterial strains: *X. aichiensis* sp. nov. XENO-7^T^, *X. bovienii* subsp. *africana* XENO-1^T^, and *X. bovienii* subsp. *bovienii* T228^T^ are *S. litorale, S. africanum,* and *S. feltiae*, respectively. These nematode species are also closely related (Fig. S6). The observed phylogenetic congruence points toward potential co-evolution between these two groups of organisms, which has been already suggested in the past [74–76]. Further studies with deeper sampling sizes will be required to conclusively test this hypothesis.

### Taxonomic Conclusions and Final Considerations

There is a strong scientific consensus around the use of dDDH values to delimit bacterial species and subspecies [57, 58, 77]. When dDDH values between two strains are below 70%, the two bacterial strains belong to different species. If their dDDH values are between 70 and 79%, the two bacterial species belong to the same species but to different subspecies. When their dDDH values are above 79%, the two bacterial strains belong to the same species and subspecies. However, when dDDH values between two bacterial strains are very close to 70% or to 79%, the taxonomic decisions are not that straightforward, and additional factors should be considered. In this study, for instance, we observed that the dDDH value between XENO-7^T^ and *X. bovienii* subsp. *bovienii* T228^T^ is 69.4%, which is very close to the species threshold, motivating the question whether XENO-7^T^ should actually be considered to represent a subspecies within the species *X. bovienii*, or a different species. The fact that dDDH value between XENO-7^T^ and *X. bovienii* subsp. *africana* XENO-1^T^ is 63.6% which indicates that XENO-7^T^ does belong to a different species, in spite that dDDH value between XENO-7^T^ and *X. bovienii* subsp. *bovienii* T228^T^ is close to the species threshold. Considering the above and based on the results of this polyphasic taxonomic study, we recommend the use of whole-genome sequences and sequence comparison methods such as the GBPD (Genome Blast Distance Phylogeny) for future description of novel *Xenorhabdus* species. Hence, the following novel species are proposed: *Xenorhabdus aichiensis* sp. nov. with XENO-7^T^ (= CCM 9233^T^ = CCOS 2024^T^) as the type strain, *Xenorhabdus anantnagensis* sp. nov. with XENO-2^T^ (= CCM 9237^T^ = CCOS 2023^T^) as the type strain, and *Xenorhabdus yunnanensis* sp. nov. with XENO-10^T^ (= CCM 9322^T^ = CCOS 2071^T^) as the type strain.

### Description of *Xenorhabdus aichiensis* sp. nov.

**Fig. 3:**
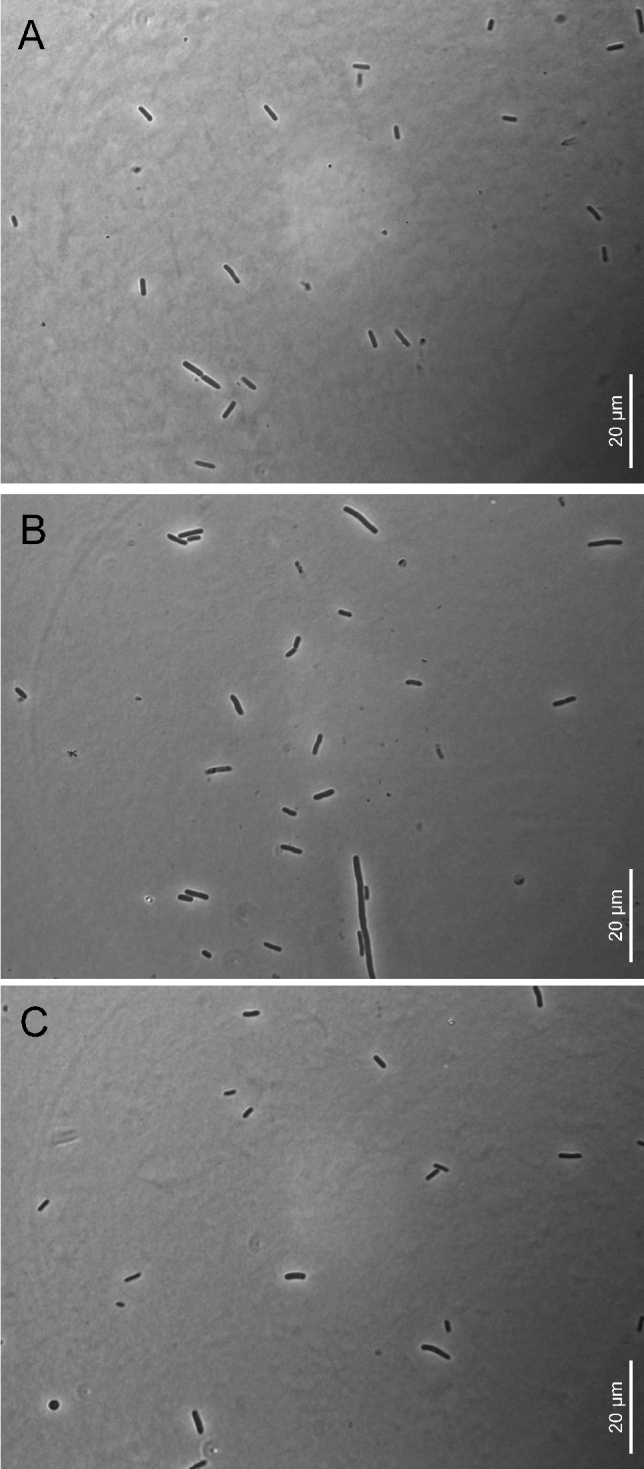
Light microscopy microphotographs of the newly described bacterial species. **a** *Xenorhabdus aichiensis* sp. nov. XENO-7^T^. **b** *Xenorhabdus anantnagensis* sp. nov. XENO-2^T^*.* **c** *Xenorhabdus yunnanensis* sp. nov. XENO-10^T^. Bars correspond to 20 µm

(ai.chi.en’sis. N.L. fem. adj. *aichiensis*, pertaining to the Aichi, Japan, the Japanese prefecture where the nematodes hosting the bacterial type strain were collected). Cells are rod-shaped, approx. 1.1–1.8 µm wide and 3.6–5.3 µm long (Fig. 3). Cells are highly pathogenic when injected in *G. mellonella* larvae. Colonies are light or dark yellow and of about 4–5 mm after 48 h on LB agar plates. When cultured in LB agar plates (pH 7, 1% NaCl), growth is observed at temperatures between 20 and 37 °C, but not at 42 °C, and optimal growth occurs at 28–30 °C. When cultured in LB (28 °C, 1% NaCl), bacterial growth occurs at pH between 5 and 9 (optimum 5–7) and not at pH 3. Bacterial growth occurs in LB medium (28 °C, pH 7.0) containing NaCl between 1 and 3% (optimum 1–2%), negative for β-galactosidase, lysine decarboxylase, ornithine decarboxylase, and tryptophan deaminase, positive for arginine dihydrolase and gelatinase, oxidase positive, catalase negative, positive for citrate utilization, does not produce hydrogen sulfide or indole, produces acetoin, oxidizes glucose but does not oxidize mannitol, inositol sorbitol, rhamnose, sucrose, melibiose, amygdalin, or arabinose. The type strain was isolated from *Steinernema litorale* nematodes. These nematodes were isolated from soil samples collected from a coastal pine forest (Cape Irago-zaki, Atsumi Peninsula, Aichi Prefecture, Honshu, Japan). The type strain of the species is XENO-7^T^ (= CCM 9233^T^ = CCOS 2024^T^). Whole-genome sequences of XENO-7^T^ are deposited in the NCBI databank under the accession number JAQRFO01 and 16S rRNA gene sequences under the accession number OQ439939. The genome assembled contains 4,699,893 bp, a G+C content of 44.63, and 4196 proteins.

### Description of *Xenorhabdus anantnagensis* sp. nov.

**Fig. 4:**
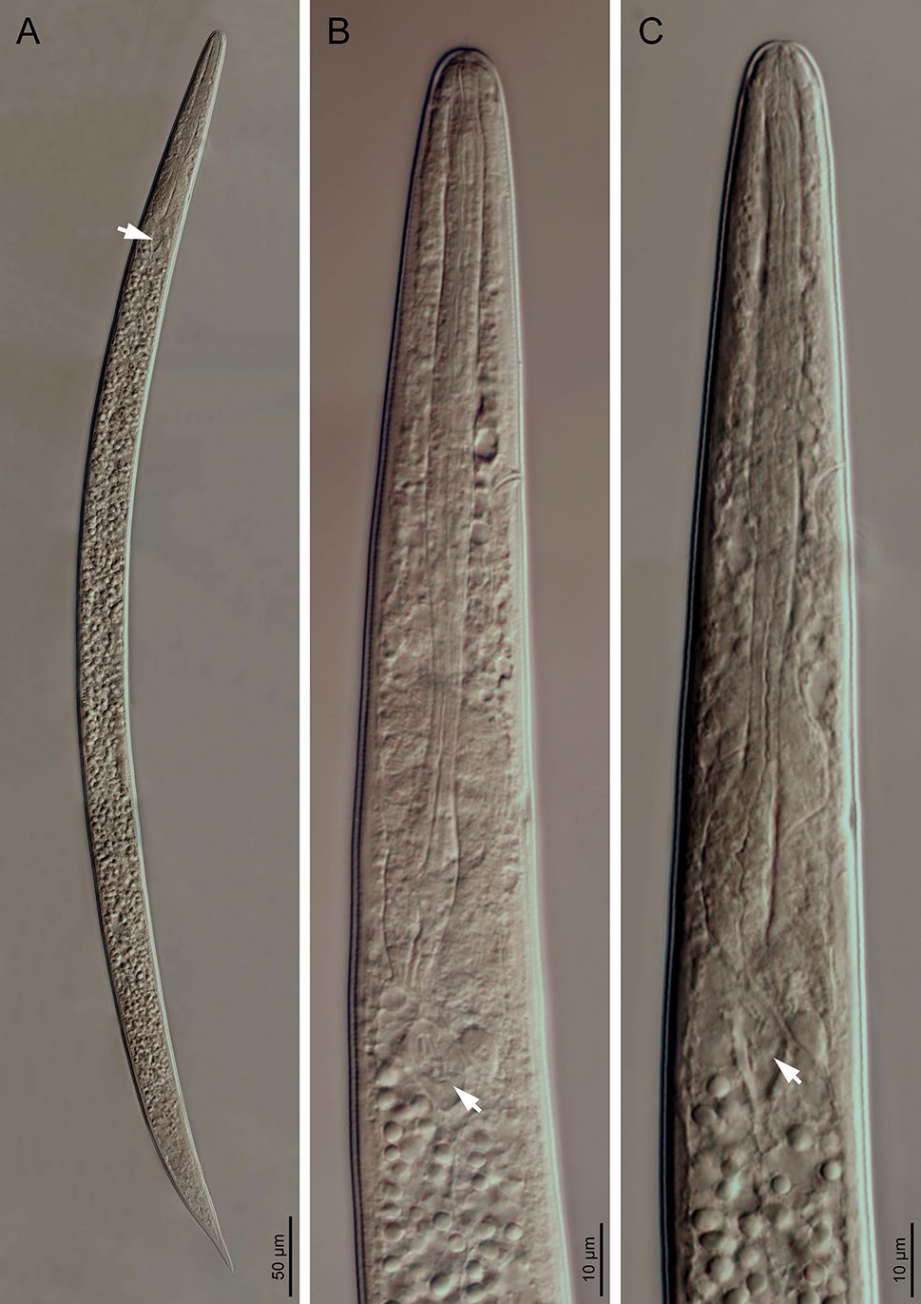
Light microscopy microphotographs of *Xenorhabdus anantnagensis* sp. nov. XENO-2^T^ within its *Steinernema* nematode host. Arrows point to the bacterial sac

(*anan.tnag.en*’*sis.* N.L. fem. adj. *anantnagensis*, pertaining to Anantnag, India, the Indian District where the nematodes hosting the bacterial type strain were collected). Cells are rod-shaped, approx. 1.2–2.1 µm wide and 4.1–6.3 µm long (Fig. 3). Cells are highly pathogenic when injected in *G. mellonella* larvae. Colonies are light or dark yellow and of about 4–5 mm after 48 h on LB agar plates. When cultured in LB agar plates (pH 7, 1% NaCl), growth is observed at temperatures between 20 and 37 °C, but not at 42 °C, and optimal growth occurs at 28–30 °C. When cultured in LB (28 °C, 1% NaCl), bacterial growth occurs at pH between 5 and 9 (optimum 5–7) and not at pH 3. Bacterial growth occurs in LB medium (28 °C, pH 7.0) containing NaCl between 1 and 3% (optimum 1–2%), negative for β-galactosidase and tryptophan deaminase, positive for arginine dihydrolase, lysine decarboxylase, ornithine decarboxylase, urease, and gelatinase activity, utilizes citrate, oxidase positive, catalase negative, produces indole, does not produce acetoin or hydrogen sulfide, oxidizes glucose but does not oxidize mannitol, inositol sorbitol, rhamnose, sucrose, melibiose, amygdalin, or arabinose. The type strain was isolated from a novel *Steinernema* species, which will be formally described elsewhere (Fig. 4). These nematodes were isolated from soil samples collected in Anantnag (Jammu and Kashmir, India) (GPS coordinates: 33.828914, 75.100091) using *Corcyra cephalonica* Stainton (Lepidoptera: Pyralidae) larvae as baits. The type strain of the species is XENO-2^T^ (= CCM 9237^T^ = CCOS 2023^T^). Whole-genome sequences of XENO-2^T^ are deposited in the NCBI databank under the accession number JAQRFN01 and 16S rRNA gene sequences under the accession number OQ439938. The genome assembled contains 4,318,764 bp, a G+C content of 42.88%, and 3905 proteins.

### Description of *Xenorhabdus yunnanensis* sp. nov.

(yun.nan.en'sis. N.L. fem. adj. *yunnanensis* pertaining to Yunnan, a province of south-west China where the nematodes hosting the bacterial type strain were collected). Cells are rod-shaped, approx. 1.5–2.0 µm wide and 3.1–6.6 µm long. Cells are highly pathogenic when injected in *G. mellonella* larvae. Colonies are light or dark yellow and of about 4-5 mm after 48 h on LB agar plates. When cultured in LB agar plates (pH 7, 1% NaCl), growth is observed at temperatures between 20 and 37 °C, but not at 42 °C, and optimal growth occurs at 28–30 °C. When cultured in LB (28 °C, 1% NaCl), bacterial growth occurs at pH between 5 and 9 (optimum 5–7) and not at pH 3. Bacterial growth occurs in LB medium (28 °C, pH 7.0) containing NaCl between 1 and 3% (optimum 1–2%), negative for β-galactosidase, lysine decarboxylase, ornithine decarboxylase, tryptophan deaminase, and urease activity, positive for arginine dihydrolase and gelatinase activity, negative for citrate utilization, oxidase and catalase negative, produces indole and acetoin, does not produce hydrogen sulfide, oxidizes glucose but does not oxidize mannitol, inositol sorbitol, rhamnose, sucrose, melibiose, amygdalin, or arabinose. The type strain was isolated from *Steinernema akhursti* nematodes. These nematodes were isolated from soil samples collected from a grassland in the Cigu village (Deqen, Diqing Tibetan Autonomous Prefecture, Yunnan province, China). The type strain of the species is XENO-10^T^ (= CCM 9322^T^ = CCOS 2071^T^). Whole-genome sequences of XENO-10^T^ are deposited in the NCBI databank under the accession number JAQRFI01 and 16S rRNA gene sequences under the accession number OQ439939. The genome assembled contains 4,667,994 bp, a G+C content of 43.14%, and 4534 proteins.

## Supplementary Information

Below is the link to the electronic supplementary material.

Supplementary file1 (PDF 462 KB)
