# Supplementary material for: Xenorhabdus aichiensis sp. nov., Xenorhabdus anantnagensis sp. nov., and Xenorhabdus yunnanensis sp. nov., Isolated from Steinernema Entomopathogenic Nematodes
Source: Curr Microbiol. 2023 Jul 26;80(9):300. doi: 10.1007/s00284-023-03373-2 (PMC10371910; doi:10.1007/s00284-023-03373-2)
Supplement: Supplementary file 1 — Supplementary file1 (PDF 462 KB) [file 284_2023_3373_MOESM1_ESM.pdf]

-SUPPLEMENTARY MATERIAL-

Xenorhabdus aichiensis sp. nov., _X. anantnagensis_ sp. nov., and _X. yunnanensis_ sp. nov., isolated from _Steinernema_ entomopathogenic nematodes

Ricardo A. R. Machado[1*], Aashaq Hussain Bhat[1,2], Carlos Castaneda-Alvarez[1,3], Tarique Hassan Askary[4], Vladimir Půža[5], Sylvie Pagès[6], Joaquín Abolafia[7]

[1]*Experimental Biology Research Group. Institute of Biology. University of Neuchâtel. Neuchâtel, Switzerland.*

[2]*Department of Biosciences. University Center for Research and Development. Chandigarh University. Gharuan, India.*

[3]*Departamento de Sanidad Vegetal. Facultad de Ciencias Agronómicas. Universidad de Chile. Santiago, Chile.*

[4]*Division of Entomology. Faculty of Agriculture. Sher-e-Kashmir University of Agricultural Sciences and Technology, Wadura Campus. Jammu and Kashmir, India.*

[5]*Biology Centre CAS. Institute of Entomology. České Budějovice, Czech Republic.*

[6]*INRAe. Université de Montpellier. Montpellier, France.*

[7]*Departamento de Biología Animal, Biología Vegetal y Ecología. Universidad de Jaén, Campus 'Las Lagunillas'. Jaén, Spain.*

[*]Corresponding author: Ricardo A. R. Machado (ricardo.machado@unine.ch). Experimental Biology Research Group. Institute of Biology. University of Neuchâtel. Rue Emile-Argand 11, 2000 Neuchâtel, Switzerland. +41 (0) 32 718 3076.

-SUPPLEMENTARY FIGURES-

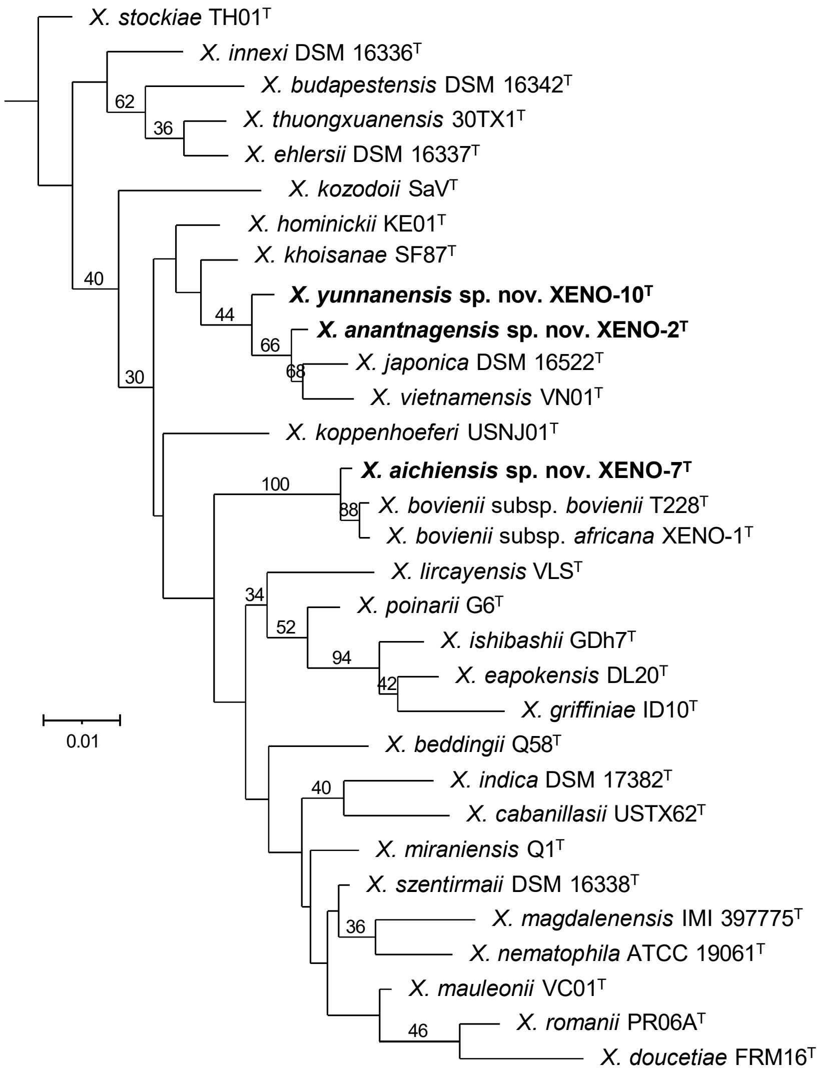

Figure S1. Maximum-likelihood phylogenetic tree of *Xenorhabdus* bacterial strains reconstructed from 1366 nucleotide positions of 16S ribosomal RNA gene sequences. Numbers at nodes represent bootstrap values based on 100 replications. Bar represent average nucleotide substitutions per sequence position. NCBI accession numbers of gene sequences used for the reconstruction are shown in Table S1

	X. stockiae TH01^{T}	*X. innexi* DSM 16336^{T}	*X. budapestensis* DSM 16342^{T}	*X. thuongxuanensis* 30TX1^{T}	*X. ehlersii* DSM 16337^{T}	*X. kozodoii* SaV^{T}	*X. hominickii* KE01^{T}	*X. khoisanae* SF87^{T}	***X. yunnanensis* sp. nov. XENO-10^{T}**	***X. anantnagensis* sp. nov. XENO-2^{T}**	*X. japonica* DSM 16522^{T}	*X. vietnamensis* VN01^{T}	*X. koppenhoeferi* USNJ01^{T}	***X. aichiensis* sp. nov. XENO-7^{T}**	*X. bovienii* subsp. *bovienii* T228^{T}	*X. bovienii* subsp. *africana* XENO-1^{T}	*X. lircayensis* VLS^{T}	*X. poinarii* G6^{T}	*X. ishibashii* GDh7^{T}	*X. eapokensis* DL20^{T}	*X. griffiniae* ID10^{T}	*X. beddingii* Q58^{T}	*X. indica* DSM 17382^{T}	*X. cabanillasii* USTX62^{T}	*X. miraniensis* Q1^{T}	*X. szentirmaii* DSM 16338^{T}	*X. magdalenensis* IM139775^{T}	*X. nematophila* ATCC 19061^{T}	*X. mauleonii* VC01^{T}	*X. doucetiae* FRM16^{T}	*X. romanii* PR6a^{T}
X. stockiae TH01^{T}	ID	98.0	97.2	96.9	97.4	96.9	97.6	97.3	96.9	97.3	97.0	97.2	96.8	96.4	96.1	96.0	96.1	96.5	96.7	96.8	95.8	96.4	97.3	96.3	96.9	97.3	96.8	96.4	97.2	96.4	96.1
X. innexi DSM 16336^{T}	98.0	ID	97.3	97.2	97.7	96.4	97.3	97.2	96.7	97.2	97.3	97.1	96.7	96.2	95.9	96.0	95.7	96.1	96.7	96.5	95.9	96.1	96.7	96.1	96.4	96.8	95.8	95.9	96.8	96.5	96.0
X. budapestensis DSM 16342^{T}	97.2	97.3	ID	97.4	98.1	96.1	97.0	97.2	96.9	96.9	96.9	96.9	96.9	96.3	96.3	96.3	95.7	95.8	96.6	96.3	95.9	95.7	96.8	96.1	96.8	96.5	96.1	95.7	96.2	96.7	96.2
X. thuongxuanensis 30TX1^{T}	96.9	97.2	97.4	ID	98.2	96.6	97.3	97.6	97.2	97.3	96.9	97.2	96.8	96.4	96.1	96.3	95.9	96.4	97.6	97.2	96.5	95.8	96.4	95.8	96.9	96.6	95.8	95.6	96.3	97.1	96.0
X. ehlersii DSM 16337^{T}	97.4	97.7	98.1	98.2	ID	96.8	97.3	97.3	96.9	97.5	97.1	97.6	96.7	96.4	96.1	96.1	95.6	96.3	97.6	97.5	97.0	95.9	96.7	95.7	97.1	96.5	96.1	95.6	96.6	97.2	96.5
X. kozodoii SaV^{T}	96.9	96.4	96.1	96.6	96.8	ID	96.7	97.5	96.6	97.1	96.5	96.7	96.4	96.2	95.8	95.8	96.3	96.1	96.5	96.1	96.3	95.8	96.5	94.8	96.2	96.2	95.8	95.6	96.1	95.7	95.6
X. hominickii KE01^{T}	97.6	97.3	97.0	97.3	97.3	96.7	ID	98.6	98.0	97.6	97.4	97.5	97.7	97.5	97.0	96.8	97.3	96.6	96.9	97.0	95.8	96.3	96.9	95.8	97.0	97.3	96.5	96.6	97.2	96.7	96.1
X. khoisanae SF87^{T}	97.3	97.2	97.2	97.6	97.3	97.5	98.6	ID	98.4	98.2	97.7	97.8	97.8	97.2	96.9	96.9	97.5	96.4	96.9	96.9	96.2	96.7	96.7	95.8	97.5	97.6	96.6	97.1	96.9	97.0	96.3
***X. yunnanensis* sp. nov. XENO-10^{T}**	96.9	96.7	96.9	97.2	96.9	96.6	98.0	98.4	ID	98.7	98.3	98.3	97.8	96.7	96.7	96.4	97.3	95.9	96.1	96.2	95.5	96.4	96.4	96.0	96.9	97.5	96.6	96.9	96.9	97.6	96.3
***X. anantnagensis* sp. nov. XENO-2^{T}**	97.3	97.2	96.9	97.3	97.5	97.1	97.6	98.2	98.7	ID	98.9	98.9	97.7	96.7	96.6	96.6	96.8	96.1	96.1	96.2	95.9	96.7	96.7	95.8	96.9	97.5	96.7	97.2	97.3	97.5	96.4
X. japonica DSM 16522^{T}	97.0	97.3	96.9	96.9	97.1	96.5	97.4	97.7	98.3	98.9	ID	98.7	97.8	96.9	96.9	96.7	96.8	96.2	96.1	96.1	95.4	96.4	96.6	96.2	96.6	97.5	96.6	97.0	97.2	97.4	96.2
X. vietnamensis VN01^{T}	97.2	97.1	96.9	97.2	97.6	96.7	97.5	97.8	98.3	98.9	98.7	ID	97.4	96.7	96.9	96.7	96.6	96.1	96.1	96.0	96.1	96.4	96.5	95.7	97.0	97.2	96.9	96.7	97.2	97.5	96.4
X. koppenhoeferi USNJ01^{T}	96.8	96.7	96.9	96.8	96.7	96.4	97.7	97.8	97.8	97.7	97.8	97.4	ID	96.6	96.8	96.4	97.1	96.1	96.3	96.0	95.4	96.1	96.8	96.3	96.5	97.7	96.3	96.7	97.0	96.8	95.9
***X. aichiensis* sp. nov. XENO-7^{T}**	96.4	96.2	96.3	96.4	96.4	96.2	97.5	97.2	96.7	96.7	96.9	96.7	96.6	ID	99.4	99.2	96.9	96.8	96.8	96.6	95.9	96.2	96.4	96.0	97.1	97.2	97.0	96.5	97.2	95.6	96.0
X. bovienii subsp. *bovienii* T228^{T}	96.1	95.9	96.3	96.1	96.1	95.8	97.0	96.9	96.7	96.6	96.9	96.9	96.8	99.4	ID	99.4	97.0	96.7	96.5	96.3	95.8	96.1	96.1	96.1	96.8	97.2	96.9	96.5	97.2	95.8	95.9
X. bovienii subsp. *africana* XENO-1^{T}	96.0	96.0	96.3	96.3	96.1	95.8	96.8	96.9	96.4	96.6	96.7	96.7	96.4	99.2	99.4	ID	96.7	96.6	96.5	96.2	95.8	96.1	96.0	96.0	96.7	97.1	96.7	96.5	97.0	95.6	95.8
X. lircayensis VLS^{T}	96.1	95.7	95.7	95.9	95.6	96.3	97.3	97.5	97.3	96.8	96.8	96.6	97.1	96.9	97.0	96.7	ID	97.3	97.2	97.2	96.7	96.9	97.0	96.6	97.1	98.0	96.9	97.2	97.8	96.0	96.5
X. poinarii G6^{T}	96.5	96.1	95.8	96.4	96.3	96.1	96.6	96.4	95.9	96.1	96.2	96.1	96.1	96.8	96.7	96.6	97.3	ID	97.7	97.7	96.7	97.5	97.0	96.4	97.0	97.4	96.7	96.8	97.2	95.3	96.5
X. ishibashii GDh7^{T}	96.7	96.7	96.6	97.6	97.6	96.5	96.9	96.9	96.1	96.1	96.1	96.1	96.3	96.8	96.5	96.5	97.2	97.7	ID	98.6	98.0	96.7	97.7	96.9	97.6	97.8	97.1	96.6	97.5	96.5	97.2
X. eapokensis DL20^{T}	96.8	96.5	96.3	97.2	97.5	96.1	97.0	96.9	96.2	96.2	96.1	96.0	96.0	96.6	96.3	96.2	97.2	97.7	98.6	ID	98.0	96.9	97.5	96.7	97.8	97.4	97.2	96.3	97.5	96.7	97.5
X. griffiniae ID10^{T}	95.8	95.9	95.9	96.5	97.0	96.3	95.8	96.2	95.5	95.9	95.4	96.1	95.4	95.9	95.8	95.8	96.7	96.7	98.0	98.0	ID	96.1	96.6	95.6	96.7	96.7	96.2	95.6	96.9	96.2	96.7
X. beddingii Q58^{T}	96.4	96.1	95.7	95.8	95.9	95.8	96.3	96.7	96.4	96.7	96.4	96.4	96.1	96.2	96.1	96.1	96.9	97.5	96.7	96.9	96.1	ID	96.9	96.9	97.5	97.5	96.9	97.1	97.2	96.0	96.9
X. indica DSM 17382^{T}	97.3	96.7	96.8	96.4	96.7	96.5	96.9	96.7	96.4	96.7	96.6	96.5	96.8	96.4	96.1	96.0	97.0	97.0	97.7	97.5	96.6	96.9	ID	97.5	97.5	97.9	97.7	96.4	97.3	95.9	97.2
X. cabanillasii USTX62^{T}	96.3	96.1	96.1	95.8	95.7	94.8	95.8	95.8	96.0	95.8	96.2	95.7	96.3	96.0	96.1	96.0	96.6	96.4	96.9	96.7	95.6	96.9	97.5	ID	97.5	97.8	97.1	96.7	97.1	95.6	96.5
X. miraniensis Q1^{T}	96.9	96.4	96.8	96.9	97.1	96.2	97.0	97.5	96.9	96.9	96.6	97.0	96.5	97.1	96.8	96.7	97.1	97.0	97.6	97.8	96.7	97.5	97.5	97.5	ID	98.5	98.1	96.9	98.0	96.1	97.5
X. szentirmaii DSM 16338^{T}	97.3	96.8	96.5	96.6	96.5	96.2	97.3	97.6	97.5	97.5	97.5	97.2	97.7	97.2	97.2	97.1	98.0	97.4	97.8	97.4	96.7	97.5	97.9	97.8	98.5	ID	98.0	98.0	98.8	96.4	97.5
X. magdalenensis IM139775^{T}	96.8	95.8	96.1	95.8	96.1	95.8	96.5	96.6	96.6	96.7	96.6	96.9	96.3	97.0	96.9	96.7	96.9	96.7	97.1	97.2	96.2	96.9	97.7	97.1	98.1	98.0	ID	97.3	97.4	96.2	97.1
X. nematophila ATCC 19061^{T}	96.4	95.9	95.7	95.6	95.6	95.6	96.6	97.1	96.9	97.2	97.0	96.7	96.7	96.5	96.5	96.5	97.2	96.8	96.6	96.3	95.6	97.1	96.4	96.7	96.9	98.0	97.3	ID	97.8	96.4	96.6
X. mauleonii VC01^{T}	97.2	96.8	96.2	96.3	96.6	96.1	97.2	96.9	96.9	97.3	97.2	97.2	97.0	97.2	97.2	97.0	97.8	97.2	97.5	97.5	96.9	97.2	97.3	97.1	98.0	98.8	97.4	97.8	ID	96.8	97.9
X. doucetiae FRM16^{T}	96.4	96.5	96.7	97.1	97.2	95.7	96.7	97.0	97.6	97.5	97.4	97.5	96.8	95.6	95.8	95.6	96.0	95.3	96.5	96.7	96.2	96.0	95.9	95.6	96.1	96.4	96.2	96.4	96.8	ID	97.8
X. romanii PR6a^{T}	96.1	96.0	96.2	96.0	96.5	95.6	96.1	96.3	96.3	96.4	96.2	96.4	95.9	96.0	95.9	95.8	96.5	96.5	97.2	97.5	96.7	96.9	97.2	96.5	97.5	97.5	97.1	96.6	97.9	97.8	ID

Figure S2. Pairwise nucleotide similarities (%) of 16S rRNA gene sequences of *Xenorhabdus* strains. 1366 nucleotide positions were analyzed. NCBI accession numbers of gene sequences used are shown in Table S1.

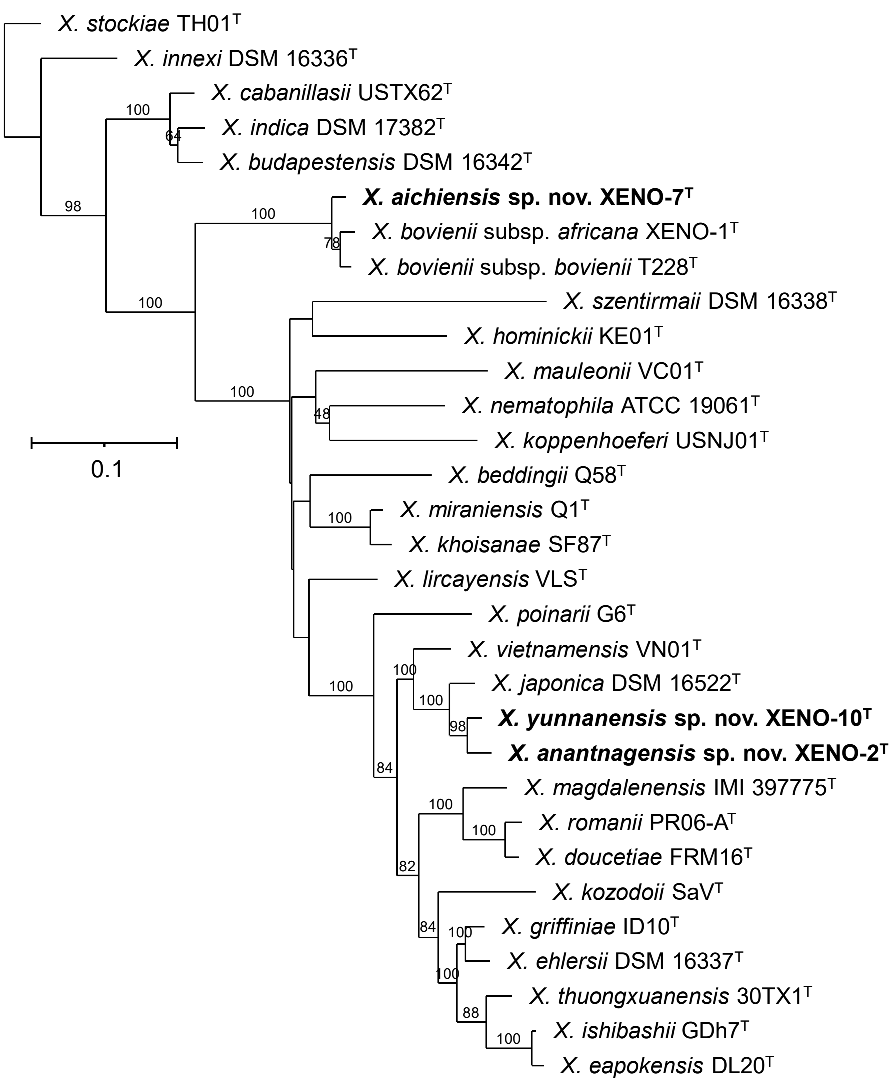

Figure S3. Maximum-likelihood phylogenetic tree of *Xenorhabdus* bacterial strains reconstructed from 1077 nucleotide positions of the recombinase A (*recA*) gene sequences. Numbers at nodes represent bootstrap values based on 100 replications. Bar represent average nucleotide substitutions per sequence position. NCBI accession numbers of gene sequences used for the reconstruction are shown in Table S1

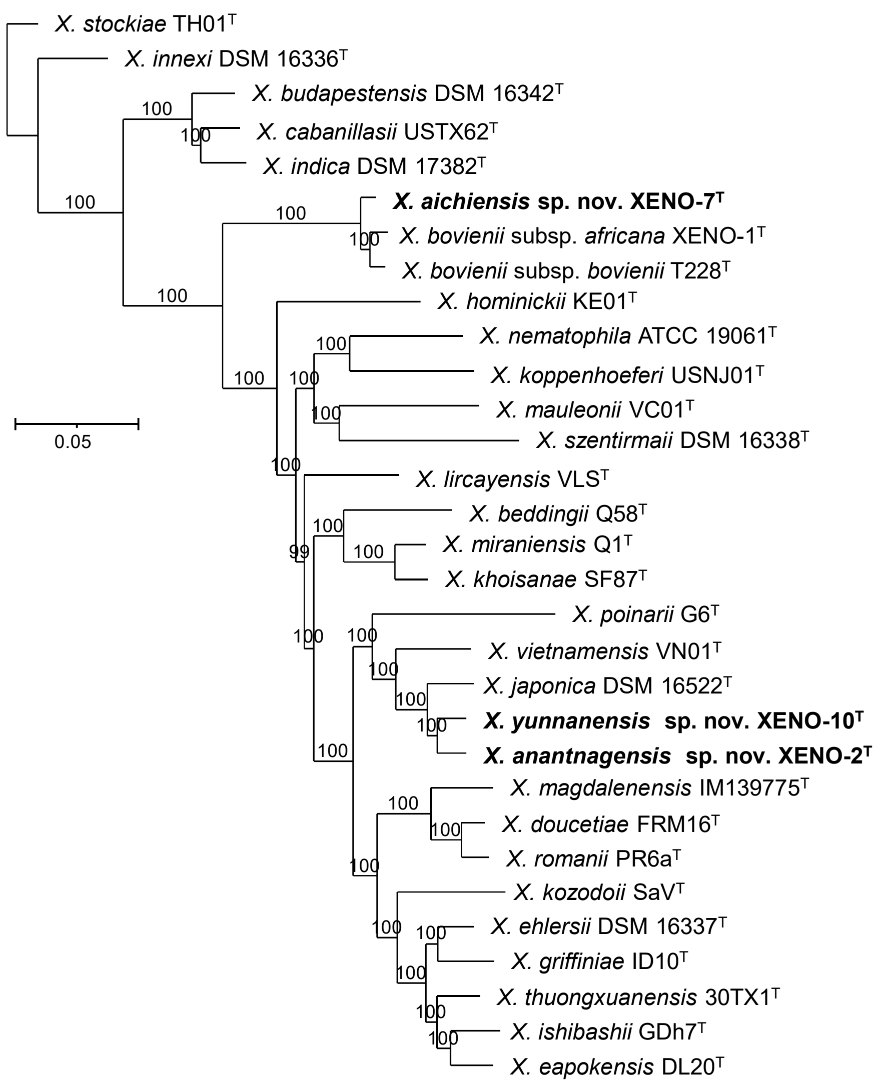

Figure S4. Phylogenetic reconstruction based on core proteome sequences of the type strains of all *Xenorhabdus* species with validly published names. A total of 1447 core proteins (495921 amino acid positions) were considered in the analyses. Phylogenetic trees were built using RAxML. Numbers at the nodes represent branch supports. Bar represents amino acid substitutions per sequence position. NCBI accession numbers of the genome sequences used are shown in Table S1.

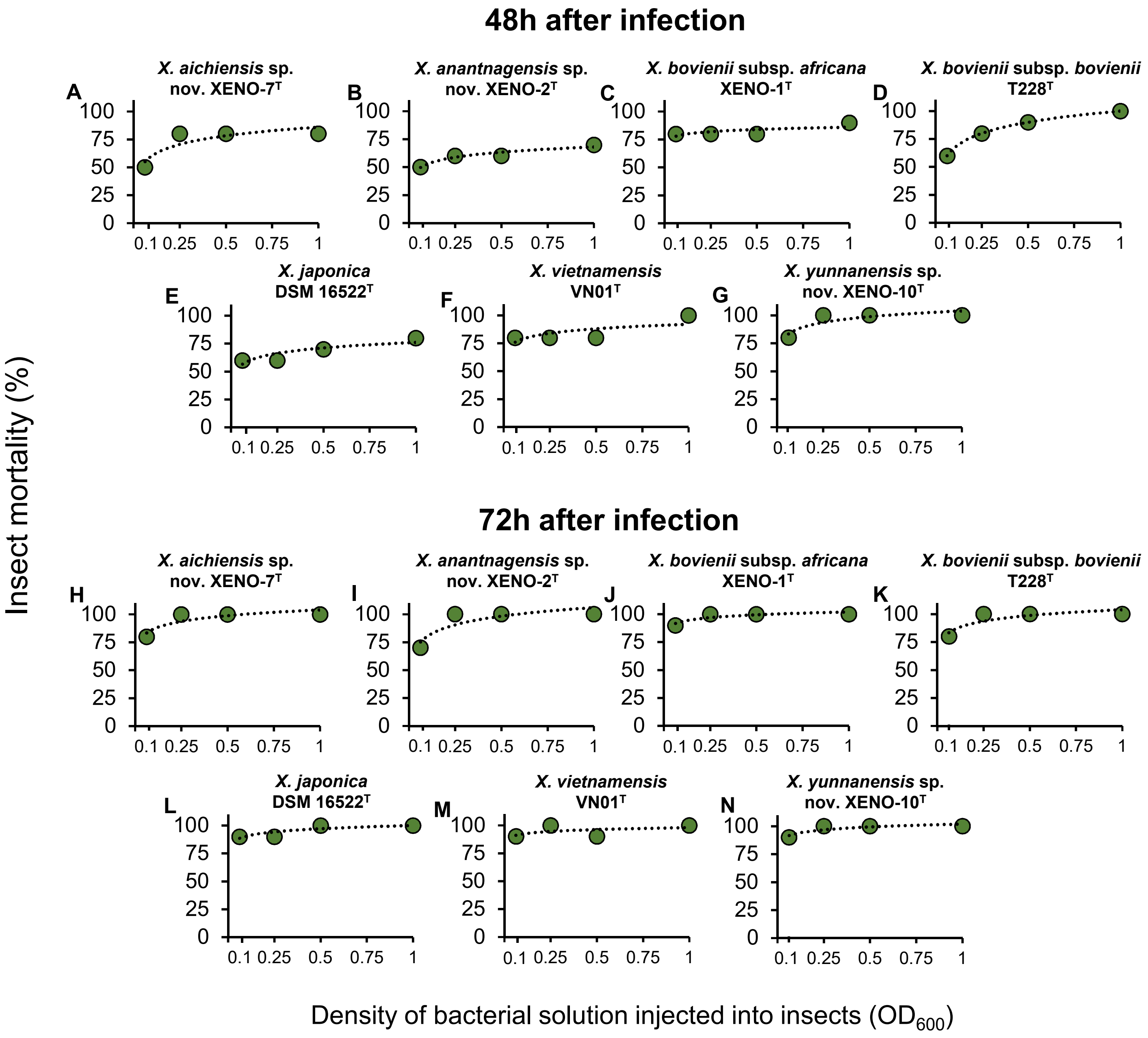

Figure S5. Entomopathogenicity of the type strains of different *Xenorhabdus* species. Insect mortality (%) caused by injecting: A, H) *X. aichiensis* sp. nov. XENO-7^T; B, I) *X. anantnagensis* sp. nov. XENO-2^T; C, J) *X. bovienii* subsp. *africana* XENO-1^T; D, K) *X. bovienii* subsp. *bovienii* T228^T; E, L) *X. japonica* DSM 16522^T; F, M) *X. vietnamensis* VN01^T; or G, N) *X. yunnanensis* sp. nov. XENO-10^T. A-G) Insect mortality (%) 48h after injecting the bacterial strains at different densities. H-N) Insect mortality (%) 72h after injecting the bacterial strains at different densities.

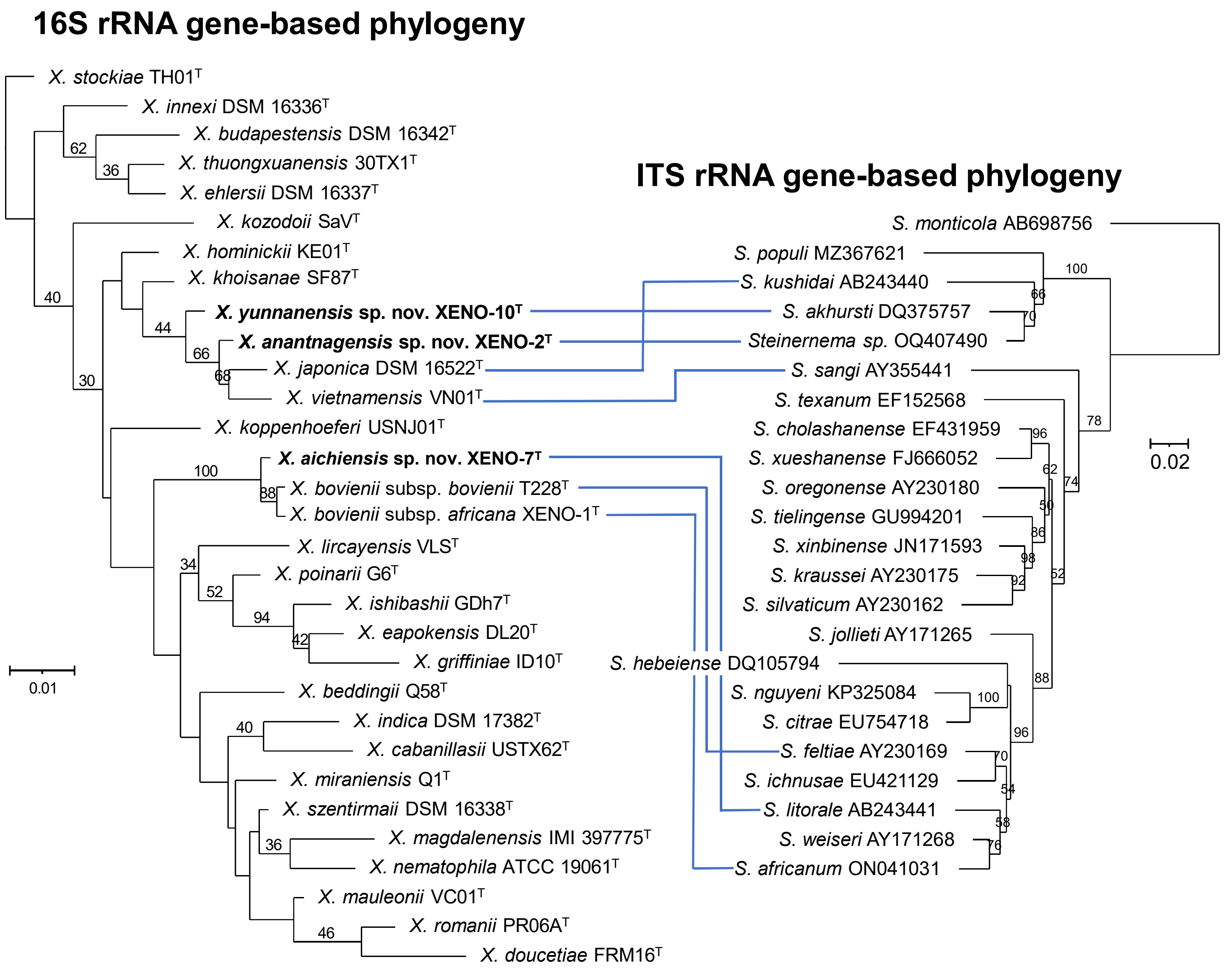

Figure S6. Tanglegram of *Xenorhabdus* and *Steinernema.* Blue lines connect the newly describe bacterial species and their more closely relates species with their respective nematode hosts. Bacterial phylogenetic relationships were reconstructed from 1366 nucleotide positions of 16S ribosomal RNA gene sequences. Nematode phylogenetic relationships were reconstructed from the sequences of the Internal Transcribed Spacer (ITS1-5.8S-ITS2) rRNA gene. Numbers at nodes represent bootstrap values based on 100 replications. Bar represent average nucleotide substitutions per sequence position. NCBI accession numbers of gene sequences used for the bacterial phylogenetic relationship reconstruction are shown in Table S1 and for nematode phylogenetic relationship reconstruction are shown on the tree.

-SUPPLEMENTARY TABLES-

Table S1. National Center for Biotechnology Information (NCBI) accession numbers of the sequences used in this study. Sequences obtained in this study are in bold.

Strain	16S	Genome
X. aichiensis **sp. nov. XENO-7^T**	**OQ439939**	**JAQRFO01**
X. anantnagensis **sp. nov. XENO-2^T**	**OQ439938**	**JAQRFN01**
X. beddingii Q58^T	AY278675	MUBK01
X. bovienii subsp. *bovienii* T228^T	AY278673	JANAIF01
X. bovienii subsp. *africana* XENO-1^T	ON497255	JAMGSK01
X. budapestensis DSM 16342^T	AJ810293	NIBS01
X. cabanillasii USTX62^T	AY521244	QTUB01
X. doucetiae FRM16^T	DQ211709	FO704550
X. eapokensis DL20^T	KX602187	MKGQ01
X. ehlersii DSM 16337^T	AJ810294	NIBT01
X. griffiniae ID10^T	DQ211710	**JAQRFM01**
X. hominickii KE01^T	DQ211719	NJAI01
X. indica DSM 17382^T	AM040494	NKHP01
X. innexi DSM 16336^T	AJ810292	NIBU01
X. ishibashii GDh7^T	GQ149086	NJAK01
X. japonica DSM 16522^T	D78008	FOVO01
X. khoisanae SF87^T	HQ142625	**JAQRFL01**
X. koppenhoeferi USNJ01^T	DQ205450	FPBJ01
X. kozodoii SaV^T	DQ211716	NJCX01
X. lircayensis VLS^T	MT466535	JACOII01
X. magdalenensis IMI 397775^T	HQ877464	**JAQRFK01**
X. mauleonii VC01^T	DQ211715	NITY01
X. miraniensis Q1^T	DQ211713	NITZ01
X. nematophila ATCC 19061^T	AY278674	FN667742
X. poinarii G6^T	D78010	FO704551
X. romanii PR06-A^T	DQ211717	**JAQRFJ01**
X. stockiae TH01^T	DQ202309	NJAJ01
X. szentirmaii DSM 16338^T	AJ810295	NIBV01
X. thuongxuanensis 30TX1^T	KX602193	MKGR01
X. vietnamensis VN01^T	DQ205447	MUBJ01
X. yunnanensis **sp. nov. XENO-10^T**	**OQ439939**	**JAQRFI01**

Table S2. Features of the genomes of the *Xenorhabdus* species generated in this study.

	***X. aichiensis* sp. nov. XENO-7^T**	***X. anantnagensis* sp. nov. XENO-2^T**	*X. griffiniae* ID10^T	*X. khoisanae* SF87^T	*X. magdalenensis* IM139775^T	*X. romanii* PR6a^T	***X. yunnanensis* sp. nov. XENO-10^T**
Scaffold L50	19	10	19	21	27	24	21
Scaffold N50	80897	157967	77915	82034	40780	47189	75444
Scaffold L90	58	33	65	71	99	81	72
Scaffold N90	23121	31679	18087	15793	8775	11496	11625
Scaffold len_max	280689	409088	203235	177774	125835	153762	195866
Scaffold len_min	500	582	508	524	509	513	200
Scaffold len_mean	31756	47986	26344	26259	14664	17715	11669
Scaffold len_median	10610	13615	7034	8353	4286	4503	719
Scaffold len_std	45506	76451	39686	38182	22046	27364	28615
Scaffold num_A	1311029	1241667	1292263	1360024	1012412	1074222	1322278
Scaffold num_T	1291258	1225138	1275115	1339984	1006374	1057662	1332102
Scaffold num_C	1063281	938828	995224	1059992	846679	879372	1004664
Scaffold num_G	1034325	913131	995032	1045416	844618	868538	1008950
Scaffold num_N	0	0	0	0	0	0	0
Scaffold num_bp	4699893	4318764	4557634	4805416	3710083	3879794	4667994
Scaffold num_bp_not_N	4699893	4318764	4557634	4805416	3710083	3879794	4667994
Scaffold num_seq	148	90	173	183	253	219	400
Scaffold GC content overall	44.63	42.88	43.67	43.81	45.59	45.05	43.14

Table S3. Features of the genomes of the *Xenorhabdus* species used in this study.

Species	Basepairs	Percent G+C	No. proteins
***X. aichiensis* sp. nov. XENO-7^{T}**	4 699 893	44.63	4196
***X. anantnagensis* sp. nov. XENO-2^{T}**	4 318 764	42.88	3905
X. beddingii Q58^{T}	4 096 354	45.19	3633
X. bovienii subsp. *africana* XENO-1^{T}	4 674 198	44.73	4217
X. bovienii subsp. *bovienii* T228^{T}	4 567 727	44.73	4395
X. budapestensis DSM 16342^{T}	4 311 148	43.13	3686
X. cabanillasii USTX62^{T}	4 335 622	42.90	3812
X. doucetiae FRM16^{T}	4 195 202	45.71	3645
X. eapokensis DL20^{T}	4 242 650	43.49	3757
X. ehlersii DSM 16337^{T}	4 058 264	43.78	3807
X. griffiniae ID10^{T}	4 557 634	43.67	4187
X. hominickii KE01^{T}	5 335 857	43.39	4871
X. indica DSM 17382^{T}	4 501 483	42.86	3800
X. innexi DSM 16336^{T}	4 573 808	43.67	4019
X. ishibashii GDh7^{T}	3 859 346	42.94	3554
X. japonica DSM 16522^{T}	3 560 854	42.70	3214
X. khoisanae SF87T	4 805 416	43.81	4322
X. koppenhoeferi USNJ01^{T}	3 181 650	43.06	2763
X. kozodoii SaV^{T}	4 127 775	44.70	3726
X. lircayensis VLS^{T}	4 279 293	44.15	3626
X. magdalenensis IM139775^{T}	3 710 083	45.59	3407
X. mauleonii VC01^{T}	5 117 864	43.90	4428
X. miraniensis Q1^{T}	4 992 355	43.67	4252
X. nematophila ATCC 19061^{T}	4 587 837	44.21	4549
X. poinarii G6^{T}	3 659 523	44.55	3371
X. romanii PR6a^{T}	3 879 794	45.05	3631
X. stockiae TH01^{T}	4 667 712	43.52	4021
X. szentirmaii DSM 16338^{T}	4 824 775	43.98	4566
X. thuongxuanensis 30TX1^{T}	3 935 399	43.05	3474
X. vietnamensis VN01^{T}	4 663 685	42.99	3909
***X. yunnanensis* sp. nov. XENO-10^{T}**	4 667 994	43.14	4534

Table S4. Completeness (%) and contamination (%) of the bacterial genomes generated in this study assessed by checkM.

Strain	Completeness (%)	Contamination (%)
X. aichiensis sp. nov. XENO-7^T	100.00	0.72
X. anantnagensis sp. nov. XENO-2^T	100.00	1.62
X. griffiniae ID10^T	100.00	1.08
X. khoisanae SF87^T	100.00	0.00
X. magdalenensis IM139775^T	99.82	0.00
X. romanii PR6a^T	100.00	1.08
X. yunnanensis sp. nov. XENO-10^T	100.00	0.54

Table S5. Antibiotic-resistance of *X. aichiensis* sp. nov. XENO-7^T, *X. anantnagensis* sp. nov. XENO-2^T, *X. yunnanensis* sp. nov. XENO-10^T, and of the type strains of their closest relative species. (+): growth in presence of the antibiotic. (-): growth reduced in presence of the antibiotic.

**Antibiotic in culturing media at a concentration of 30 mg/l**	***X. aichiensis* sp. nov. XENO-7^T**	***X. anantnagensis* sp. nov. XENO-2^T**	*X. bovienii* subsp. *africana* XENO-1^T	*X. bovienii* subsp. *bovienii* T228^T	*X. japonica* DSM 16522^T	*X. poinarii* G6^T	*X. vietnamensis* VN01^T	***X. yunnanensis* sp. nov. XENO-10^T**
Tetracyclines (Tetracycline)	-	-	-	-	+	+	+	-
Aminoglycosides (Gentamicin)	+	+	+	+	+	+	+	+
Glycopeptides (Vancomycin)	+	+	-	+	+	+	+	+
